# Single-Port Laparoscopy, NOTES, and Endoluminal Surgery

**DOI:** 10.1155/2010/710130

**Published:** 2010-06-24

**Authors:** Pedro F. Escobar, Jihad H. Kaouk, Daniel Geisler, Matthew Kroh, Amanda Nickles Fader, Tommaso Falcone

**Affiliations:** ^1^Minimally Invasive Surgery and Robotics-Section of Gynecologic Oncology, Cleveland Clinic, A81, 9500 Euclid Avenue, Cleveland, OH 44195, USA; ^2^Center for Laparoscopic and Robotic Surgery, Glickman Urological and Kidney Institute, Cleveland Clinic, Q10-1, 9500 Euclid Avenue, Cleveland, OH 44195, USA; ^3^Department of Colorectal Surgery, Cleveland Clinic, A30, 9500 Euclid Avenuenue, Cleveland, OH 44195, USA; ^4^Bariatric Surgery Institute, Cleveland Clinic, A100, 9500 Euclid Avenue, Cleveland, OH 44195, USA; ^5^Department of Obstetrics and Gynecology, Johns Hopkins Medical Center and Greater Baltimore Medical Center, Baltimore, MD, USA; ^6^Cleveland Clinic Lerner College of Medicine, Chairman, OB/GYN and Women's Institute, Cleveland Clinic, A81, 9500 Euclid Avenue, Cleveland, OH 44195, USA

Minimally invasive surgery has become the standard treatment for many disease processes. In the last decade, numerous studies have demonstrated that laparoscopic approaches to various conditions have improved the quality of life with comparable surgical and oncologic outcomes to standard open procedures. Recently, an alternative to conventional laparoscopy or robotic surgery has been developed: laparoendoscopic single-site surgery (LESS), also known as single-port surgery. Single-port laparoscopy is an attempt to further enhance the cosmetic benefits of minimally invasive surgery while minimizing the potential morbidity associated with multiple incisions. In this special issue, we will focus on 2 articles relevant to the advancement and practice of LESS. 

This new modality presents unique challenges, such as instrument crowding and the need to move and control a flexible camera and articulating instruments together, requiring even more advanced laparoscopic skills. Information about training and education on single-port laparoscopy is scarce in the current literature: Ramalingam et al. present their initial experience and methodology in training and education regarding this new technique. Formal training in this technique is not widely available. Expensive ports and instrumentation may be factors deterring the training. The authors address a major gap in the literature, how to train surgeons in this technique? They modified the standard laparoscopic endotrainer with improvised ports, to make it suitable for single-port laparoscopic training. For the animal lab training, they improvised ports and low-cost instruments. Thus, the overall cost of the training in LESS was reduced, and better confidence levels were achieved prior to human applications. Perhaps this will stimulate others to look at adopting this relatively low-tech technique for training in LESS. 

Emerging data on LESS has been reported in general surgical, gynecologic, and urologic procedures. LESS, for renal surgery, was first reported in 2007, and, since then, a handful of authors have described variations of their techniques. Derweesh and coauthors present the first prospective report in the literature of LESS radical nephrectomy with renal vein thrombectomy and cytoreductive nephrectomy. As such, this report further corroborates that increasingly complex procedures can be safely performed with the LESS platform. The feasibility of LESS for complex surgical procedures is no longer an issue. Current research and development in single-port robotics is ongoing. A single-port robotic platform may overcome technical limitations of single-site surgery (instrument crowding, lack of triangulation, and loss of depth of perception/instability with current 2D flexible optics). More importantly, it may play an essential role in the reproducibility and diffusion of LESS. Prospective studies are needed to assess the relative benefits of LESS compared with more conventional minimally invasive approaches. 

*Pedro F. Escobar* *Pedro F. Escobar* *Jihad H. Kaouk* *Jihad H. Kaouk* *Daniel Geisler* *Daniel Geisler* *Matthew Kroh* *Matthew Kroh* *Amanda Nickles Fader* *Amanda Nickles Fader* *Tommaso Falcone* *Tommaso Falcone*

